# Design of Multi-Functional Bio-Safe Dental Resin Composites with Mineralization and Anti-Biofilm Properties

**DOI:** 10.3390/jfb15050120

**Published:** 2024-04-30

**Authors:** Jiaojiao Yun, Michael F. Burrow, Jukka P. Matinlinna, Hao Ding, Sin Man (Rosalind) Chan, James K. H. Tsoi, Yan Wang

**Affiliations:** 1 Department of Prosthodontics, Guanghua School of Stomatology, Hospital of Stomatology, Guangdong Provincial Key Laboratory of Stomatology, Sun Yat-sen University, Guangzhou 510055, China; yuanjj25@mail.sysu.edu.cn; 2 Dental Materials Science, Division of Applied Oral Sciences and Community Dental Care, Faculty of Dentistry, The University of Hong Kong, Hong Kong SAR, China; jpmat@hku.hk (J.P.M.); dinghao@connect.hku.hk (H.D.); chan66@connect.hku.hk (S.M.C.); 3 Prosthodontics, Division of Restorative Dental Sciences, Faculty of Dentistry, The University of Hong Kong, Hong Kong SAR, China; mfburr58@hku.hk; 4 Division of Dentistry, School of Medical Sciences, University of Manchester, Manchester M13 9PL, UK

**Keywords:** UDMA, resin composite, bioactive glass, biocompatibility, anti-biofilm activity

## Abstract

This study aims to develop multi-functional bio-safe dental resin composites with capabilities for mineralization, high in vitro biocompatibility, and anti-biofilm properties. To address this issue, experimental resin composites consisting of UDMA/TEGDMA-based dental resins and low quantities (1.9, 3.8, and 7.7 vol%) of 45S5 bioactive glass (BAG) particles were developed. To evaluate cellular responses of resin composites, MC3T3-E1 cells were (1) exposed to the original composites extracts, (2) cultured directly on the freshly cured resin composites, or (3) cultured on preconditioned composites that have been soaked in deionized water (DI water), a cell culture medium (MEM), or a simple HEPES-containing artificial remineralization promotion (SHARP) solution for 14 days. Cell adhesion, cell viability, and cell differentiation were, respectively, assessed. In addition, the anti-biofilm properties of BAG-loaded resin composites regarding bacterial viability, biofilm thickness, and biofilm morphology, were assessed for the first time. In vitro biological results demonstrated that cell metabolic activity and ALP expression were significantly diminished when subjected to composite extracts or direct contact with the resin composites containing BAG fillers. However, after the preconditioning treatments in MEM and SHARP solutions, the biomimetic calcium phosphate minerals on 7.7 vol% BAG-loaded composites revealed unimpaired or even better cellular processes, including cell adhesion, cell proliferation, and early cell differentiation. Furthermore, resin composites with 1.9, 3.8, and 7.7 vol% BAG could not only reduce cell viability in *S. mutans* biofilm on the composite surface but also reduce the biofilm thickness and bacterial aggregations. This phenomenon was more evident in BAG7.7 due to the high ionic osmotic pressure and alkaline microenvironment caused by BAG dissolution. This study concludes that multi-functional bio-safe resin composites with mineralization and anti-biofilm properties can be achieved by adding low quantities of BAG into the resin system, which offers promising abilities to mineralize as well as prevent caries without sacrificing biological activity.

## 1. Introduction

Repairing tooth defects with dental resin composite is currently the most utilized approach in clinical settings because of its tooth-colored aesthetics, minimal preparation procedure, direct-filling technique, and good mechanical characteristics [1]. However, numerous studies have shown that composite resins are more prone to accumulating plaque and biofilms on their surface than on the tooth surface and other restorative materials [2]. As a result, biofilm accumulation is usually observed at the interface between the tooth and the restoration, which can lead to secondary caries, shorten the lifespan of restorations, and eventually cause the failure of restorative composites. Therefore, formulating resin composites with antibacterial properties to inhibit bacteria, combat biofilm attachment to restoration surfaces, and prevent recurrent caries is in high demand.

During the preceding decade, to prevent secondary caries, various attempts have been performed to integrate antibacterial agents, such as chlorhexidine (CHX) [3], and nanoparticles (NP), like silver (Ag) and zinc oxide (ZnO) [4], into resin composites. These approaches could reduce the biochemical hydrolysis of resin composites catalyzed by acids or enzymes from bacteria [5]. However, the composites with these directly mixed agents showed an initial burst release. Furthermore, the release of these agents may cause structural destruction and impair the mechanical properties of the resin composites [6], which ultimately has a significant impact on the durability of composites. Recently, synthetic bioactive glass (BAG), a promising material for regenerative medicine due to its favorable biological properties e.g., biocompatibility, osteoinduction, and anti-bacterial properties, has been utilized as an additive to functionalize resin composites or orthodontic resin [7,8,9]. Our previous research [7] demonstrated biomimetic calcium phosphate formation on 45S5 BAG-loaded dental resin composites during the ion release, which may seal and minimize the marginal gaps between the tooth and composite materials, thereby helping to prevent secondary tooth decay. This specific procedure will extend the lifespan of the restorative composites. In addition, the pH increase [10,11] and osmotic pressure caused by the release of ions [11] during the dissolution of BAG in an aqueous environment are also factors that contribute to the antibacterial activity of BAG in resin composites. It has recently been reported that 45S5 BAG particles appear to be as effective as S53P4 and are possibly even more capable of limiting bacterial infections [10]. Furthermore, the efficacy of 45S5 BAG was not confined to inhibiting planktonic development, as it also extended to bacterial biofilms. It is important to emphasize that the prevention of biofilm formation on resin composites is of high scientific and clinical interest since biofilm formation contributes to the chemical and mechanical degradation of resin composites [12]. Furthermore, bacteria in biofilms are more resistant to antibacterial treatments compared to those in the planktonic state. However, no study to date has been undertaken that focuses on the anti-biofilm possibilities of BAG-containing resin composites. Doing so could help better understand the processes underlying its antibacterial effects and broaden the application of BAG in dentistry.

In addition, when BAG particles come into contact with biological fluids, the potential pH and ion-dependent cytotoxicity caused by BAG particle dissolution and degradation must be considered [13,14]. In a previous investigation [15], low quantities (8 wt%) of 45S5 BAG loading did not impair the cell metabolic activity; however, increasing BAG levels to 16 and 23 wt% diminished cell viability when utilizing undiluted composite extracts. Furthermore, cell viability became normal after adjusting the alkaline pH of composite extracts to neutral. This finding demonstrates that the main cause of the cytotoxic effects of BAG-loaded resin composites was the elevation of the surrounding pH in a dose-dependent manner. However, this non-realistic pH shift may not be observed in vivo due to in vivo dynamic circumstances that allow the leached ions to be continuously diluted [16,17]. Therefore, the preconditioning treatment of BAG materials, such as being presoaked in a variety of preconditioning solutions, including simulated body fluid (SBF) [18], tissue culture medium [19], and cell culture medium [20,21] to simulate in vivo conditions, has been put forward over the years to test the biocompatibility. However, few research studies have included this while investigating the in vitro biological reactions of BAG-loaded resin composites, even for conventional resin composites. Therefore, there is excitement over the in vivo biological response of these “bio-safe” resin composites, as well as the necessity to assess their biocompatibility in order to identify whether they are bioactive materials.

Accordingly, this study aimed to develop multi-functional bio-safe UDMA-based dental resin composites. In vitro biocompatibility of low quantities (1.9, 3.8, and 7.7 vol%) of 45S5 BAG-loaded resin composites was assessed under different laboratory protocols, including exposure to resin extracts, direct contact with the resins, and preconditioning treatments to simulate in vivo condition. Furthermore, the antibacterial effects of BAG-loaded resin composites against *S. mutans* activity in biofilm, together with anti-biofilm potential, namely biofilm thickness, bacterial aggregation, and biofilm morphology were identified using CLSM and SEM.

## 2. Materials and Methods

### 2.1. Preparation of Resin Composites

The UDMA/TEGDMA-based resin composite was prepared according to our previous study [7]. Specifically, a resin mixture of UDMA/TEGDMA (49:49 *w*/*w*) (Esstech Inc., Essington, PA, USA) was combined with the photoinitiators, including 1 wt% camphorquinone (CQ) and 1 wt% (dimethylamino)ethyl methacrylate (DMAEMA) (Esstech Inc., Essington, PA, USA), homogeneously. For the experimental resin composites, three quantities of the BAG fillers were used, namely, 1.9, 3.8, or 7.7 vol% of 45S5 bioactive glass (D_50_ = 4 μm, density 2.5–2.6 g/cm^3^, Cera Dynamics Ltd., Stoke-on-Trent, UK). The different BAG filler contents were loaded into the resin matrix with the commercial silanized BaO-Al_2_O_3_-SiO_2_-B_2_O_3_-F glass filler (DF11, D_50_ = 0.7 μm, density 2.5–2.6 g/cm^3^, Cera Dynamics Ltd., Stoke-on-Trent, UK), and contributed to the total filler load, up to 25 vol% (~65 wt%) as the control composite. All constituents were placed inside a glass beaker, which was covered with aluminum foil to protect the resin composites from ambient light, and then hand-mixed homogenously with a spatula for 5 min. After the preparation, they were stored in aluminum-foil-wrapped syringes at 4 °C before use. Disk-shaped specimens (diameter = 6.0 mm, thickness = 1.0 mm) were made and designated as BAG0, BAG1.9, BAG3.8, and BAG7.7 based on their BAG contents. All the specimens were sterilized by UV radiation for 30 min on each side in a cell culture hood.

### 2.2. Determination of Cellular Activity

Mouse calvarial pre-osteoblastic cells (MC3T3-E1, Subclone 4, CRL-2593 ™, ATCC, Manassas, VA, USA) were incubated in α-MEM (Gibco, Paisley, UK), 10% fetal bovine serum (Gibco, Paisley, UK), and 1% penicillin/streptomycin (Gibco, Paisley, UK) in a humidified atmosphere of 95% air and 5% CO_2_ at 37 °C.

#### 2.2.1. Composites Extract Group

The extracts of composites were prepared by soaking sterilized disk-shaped specimens (diameter = 6.0 mm, thickness = 1.0 mm) in 5 mL of cell culture media for 3 days, 7 days, and 14 days, respectively, as described in our previous work [7]. To evaluate the effects of the 3-day, 7-day, and 14-day extracts of experimental resin composites on cell viability, the MC3T3-E1 cells were cultured in 96-well plates, seeded at a density of 2 × 10^4^ cells/mL, and cultured with 100 μL cell culture media. After 24 h, the cells were treated with 100 μL of extracts. Cells treated with original culture media during the whole period were set as the control group. After 1, 3, and 5 days, the cells were treated with Cell Counting Kit-8 (CCK-8, APExBIO, Boston, MA, USA) culture media according to the manual. Cell viability was measured using a microplate reader (SpectraMax M2, Molecular Devices, San Diego, CA, USA) at the absorbance wavelength of 450 nm. The cell viability in the control group was set at 100%, and in the comparison groups, the changes were given as a percentage of control.

For the assessment of ALP activity, cells were differentiated in the osteogenic medium that was additionally supplemented with 50 μg/mL vitamin C, 0.01 M β-glycerophosphate, and 10^−7^ M dexamethasone. For the experimental groups, the aforementioned components were added to the composite extracts. MC3T3-E1 cells were cultured in 48-well plates and seeded at a density of 5 × 10^4^ cells/mL. After 24 h, the cells were cultured with different osteogenic differentiation media, with the media being replaced every two to three days. After 7 days, the cell lysate was processed using an ALP assay kit (Abcam, Cambridge, UK). The ALP activity was normalized by measuring the total protein content from a Pierce™ BCA Protein Assay Kit (Thermo Scientific™, Waltham, MA, USA), using bovine serum albumin (BSA, Sigma-Aldrich, Saint Louis, MO, USA) as the standard.

#### 2.2.2. Freshly Cured Composite-Direct Contact Group

The MC3T3-E1 cells were seeded on tissue culture plates (TCPS), BAG0, BAG1.9, BAG3.8, and BAG7.7 at a density of 5 × 10^4^ cells/mL and cultured with 300 μL cell culture media. Cell viability and cell differentiation evaluations were measured as shown in Section 2.2.1.

#### 2.2.3. One-Step Preconditioned Composite-Direct Contact Group

To simulate the in vivo condition to evaluate the cellular behavior, the cells were seeded on BAG7.7 and BAG7.7 after 14 days of preconditioning in deionized water (DI water), cell culture medium (MEM), and a novel Simple HEPES-containing artificial remineralization promotion (SHARP) solution (BAG7.7_DI water and BAG7.7_SHARP, respectively) based on our prior study [7], while those cultured-on tissue culture plates (TCPS) acted as positive controls. After 6 h, nuclei, F-actin, and vinculin of adherent cells were stained by DRAQ5^TM^ solution (Thermo Scientific™, Waltham, MA, USA), rhodamine phalloidin (Invitrogen™, Thermo Fisher Scientific, Waltham, MA, USA), primary anti-vinculin antibody (Invitrogen™, Thermo Fisher Scientific, Waltham, MA, USA), and Goat anti-Rabbit Alexa Fluor 488 secondary antibody (Invitrogen™, Thermo Fisher Scientific, Waltham, MA, USA), and then observed using a confocal laser scanning microscopy (CLSM, Olympus FV1000-IX81, Tokyo, Japan). Cell proliferation and cell differentiation evaluations were conducted as described in Section 2.2.1.

### 2.3. Anti-Biofilm Role of Bioactive Glass in Dental Resin Composites

#### 2.3.1. Preparation of *Streptococcus mutans*

A standard suspension of *S. mutans* (ATCC^®^ 35668™, ATCC, Manassas, VA, USA) was used as the test strain. The bacteria suspension in brain heart infusion (BHI) broth supplemented with 1% sucrose was placed in a polystyrene spectrophotometry cuvette (Sarstedt, Nümbrecht, NRW, Germany) and counted in a spectrophotometer (DU 730 UV/Vis Spectrophotometer, Beckman Coulter, Brea, CA, USA). The spectrophotometer had to measure an optical density of 0.166–0.17 at a wavelength of 660 nm, which corresponds to a concentration of 1.0 × 10^8^ CFU/mL. These suspensions were further diluted in BHI broth to obtain a working concentration of about 1.0 × 10^6^ CFU/mL.

#### 2.3.2. Growth of *S. mutans* Biofilm on Composites

The freshly cured specimens were placed inside separate wells of a 48-well tissue culture plate, to which 400 μL of the bacterial suspension was added. All samples were then incubated anaerobically at 37 °C for 1 and 3 days for biofilm maturation. The BHI broth was replaced daily during the overall incubation.

#### 2.3.3. XTT Metabolic Assay for Cellular Viability in Biofilms

After the desired time, the specimens were removed into a new 48-well tissue culture plate, followed by washing with PBS gently. The XTT reagent (Cayman Chemical, Ann Arbor, MI, USA) was added to the specimens and incubated at 37 °C for 3 h. Following this, 100 µL of supernatant was dispensed in a 96-well plate to measure absorbance at 450 nm using a microplate reader (SpectraMax M2, Molecular Devices, San Diego, CA, USA).

#### 2.3.4. CFU Counting for Cellular Viability in Biofilms

After the desired time, the formed biofilms on the resins were harvested in 1 mL of fresh BHI broth in an Eppendorf tube using a cell scraper and mixed by vortex agitation for 15 min. To inoculate the bacteria, 50 μL of diluted cell suspension was sucked into the spiral plating machine (Autoplate 4000, Spiral Biotech, Bethesda, MD, USA) and then inoculated over horse blood agar plates. All agar plates were incubated at 37 °C for 48 h in an anaerobic chamber. CFU counts of 30 to 300 were obtained for each specimen in each group.

#### 2.3.5. Live/Dead Assay for Staining

A live/dead bacterial viability kit (BacLight™, molecular probe, Invitrogen, Thermo Fisher Scientific, Waltham, MA, USA) was used to stain viable bacteria in green and dead bacteria in red, followed by the analysis using a CLSM (Olympus FV1000-IX81, Tokyo, Japan). In order to explore the cell viability distribution in the biofilm throughout its depth, the distribution of live and dead bacteria in each layer was analyzed from 2D images using ImageJ software (v1.52q, NIH, Bethesda, MD, USA), which could show the %Area values. Then the total bacterial area, live bacteria area, and dead bacteria area were plotted vs. the location of the 2D image in the biofilm at a distance from the specimen surface. The area under the curves representing bacterial volume and the peak position of the curves were assessed using GraphPad Prism software (v9.0.2, San Diego, CA, USA).

#### 2.3.6. SEM Analysis for Surface Morphology of Biofilm

SEM (SU1510, Hitachi, High-Technologies Corporation, Tokyo, Japan) was utilized to visualize the morphological features of the *S. mutans* biofilms. The resin composite discs with biofilm were fixed in 2.5% glutaraldehyde for 2 h, followed by progressive dehydration through an ascending series of ethanol solutions (70%, 85%, 95%, and 100%), air drying, and gold spraying. The images were taken at 3 randomly selected fields of view at 500× magnification. Image J software (v1.52q, NIH, Bethesda, MD, USA) was further utilized to analyze the size of bacterial clumps.

### 2.4. Statistical Analysis

Data were statistically analyzed using SPSS software (v26, SPSS Inc., Chicago, IL, USA). The normal distribution of all data was checked. One-way ANOVA with a Bonferroni post hoc test was used to determine the differences among different groups when equal variances were confirmed. If the variances were unequal, Dunnett’s T3 test was used as a post hoc test. *p* < 0.05 was considered to be statistically significant.

## 3. Results

### 3.1. Cellular Activity of BAG-Loaded Resin Composites

#### 3.1.1. Composite Extract Group

**Figure 1:**
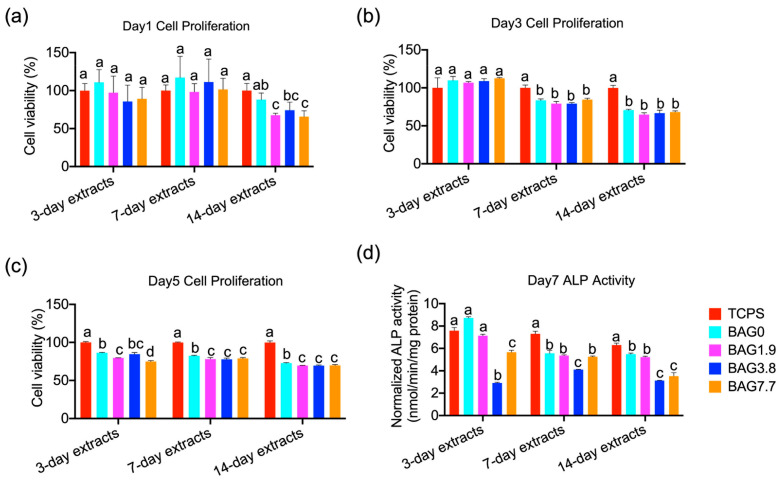
The effects of the 3-day, 7-day, and 14-day extracts of resin composites on the cell proliferation evaluated on (**a**) day 1, (**b**) day 3, (**c**) day 5, and (**d**) ALP activity on day 7, compared with the control group (no resin composites extracts) (different small letters indicate significant differences between BAG groups using extracts from the same day).

As shown in Figure 1, the day-1 cell viability of the BAG1.9, BAG3.8, and BAG7.7 groups was significantly lower than those of the TCPS and BAG0 control groups when using 14-day extracts. There was no significant change identified between BAG composites under 3-day and 7-day extracts. On day 3, the metabolic activity of the cells significantly decreased when subjected to 7-day extracts and 14-day extracts of composites, independent of the BAG amounts. All extracts from all experimental BAG-loaded resin composites had significant negative effects on cell viability on day 5. Figure 1d depicts ALP expression in cells grown in composite extractions on day 7. The 3-day extracts of BAG3.8 and BAG7.7 significantly reduced ALP expression compared to the control group (*p* < 0.05), with BAG3.8 showing the lowest values, which was also detected in the 7-day extracts. In addition, when cells were cultured in the 14-day extracts of experimental composites, all composites demonstrated adverse effects on ALP activity, compared to TCPS without extracts. The effects were more noticeable in BAG3.8 and BAG7.7 than in BAG0 and BAG1.9.

#### 3.1.2. Freshly Cured Composite-Direct Contact Group

**Figure 2:**
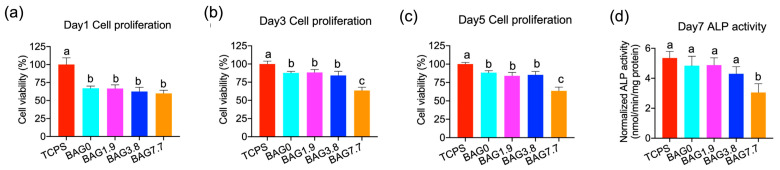
The MC3T3-E1 cell proliferation on experimental resin composites was evaluated on (**a**) day 1, (**b**) day 3, (**c**) day 5, and (**d**) ALP activity on day 7, compared with the TCPS group. (Different small letters indicate significant differences between BAG groups on the same-day cell culture).

Figure 2a–c indicate that all freshly cured resin composites had negative influences on cell viability at all time points (*p* < 0.05) when compared with TCPS. The metabolic activity of the cells was significantly reduced due to exposure to the freshly cured BAG7.7 group on day 3 and day 5 (*p* < 0.05). There was no statistical difference between BAG0, BAG1.9, and BAG3.8 (*p* > 0.05). As shown in Figure 2d, there was a decrease in ALP expression with the increase in the BAG fillers in a concentration-dependent manner. However, the change in ALP expression was statistically significant only for the BAG7.7 group, which showed the smallest ALP activity of 3.05 ± 0.59 (nmol/min/mg protein) (*p* < 0.05), compared to other groups.

#### 3.1.3. Preconditioned Composite-Direct Contact Group

**Figure 3:**
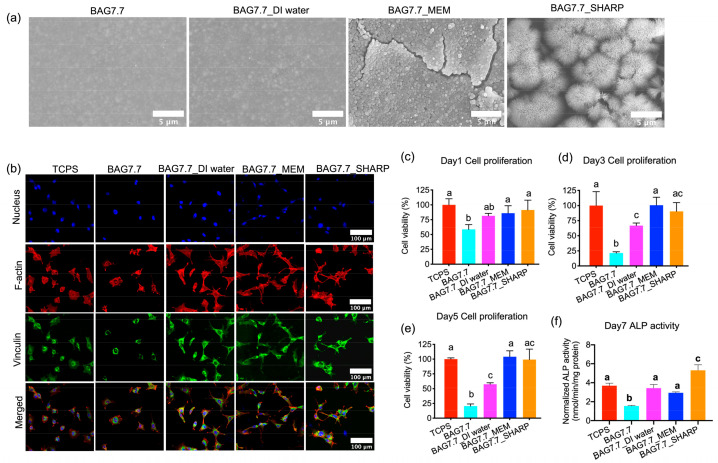
In vitro biocompatibility of biomimetic calcium phosphate layers on preconditioned resin composites. (**a**) SEM images of preconditioned composites, (**b**) immunofluorescent images of MC3T3-E1 cells after 6 h of culture. MC3T3-E1 cell proliferation on (**c**) day 1, (**d**) day 3, and (**e**) day 5. (**f**) MC3T3-E1 ALP expression on day 7. Different small letters indicate significant differences between BAG groups on the same day.

In order to further evaluate the biocompatibility of the biomimetic calcium phosphate layers of BAG-containing composites after preconditioning, the cellular activity of preconditioned composites was assessed. As shown in Figure 3a, some single and clustered spherical-shaped particles, about 300–400 nm in diameter, were observed on the surface of the preconditioned specimens in MEM, while microspheres self-assembled by nano-sized rods, with a width of 100 nm and a length of 8 μm, were formed on preconditioned composites in SHARP solutions, which were revealed as octacalcium phosphate (OCP) and carbonated-containing hydroxyapatite (CHA) crystals in our previous research [7]. Cell staining results show that MC3T3-E1 cells cultured on BAG7.7 were smaller than those cultured in other groups (Figure 3b). In contrast, the cells adherent to BAG7.7_DI water, BAG7.7_MEM, and BAG7.7_SHARP were larger and showed a cytoskeleton with distinctive stress fibers in their cytoplasm. More vinculin green spots were observed at the cytoplasm and at the end of the cellular pseudopodia on BAG7.7_DI water, BAG7.7_MEM, and BAG7.7_SHARP. Furthermore, the cell viability on BAG7.7 showed the lowest value of 58.6 ± 8.0% on day 1 (Figure 3c). On day 3, there were 75% more cells on TCPS, BAG7.7_MEM, and BAG7.7_SHARP and 50% more cells on BAG7.7_DI water in comparison to the cellular activity on BAG7.7 (Figure 3d). Similarly, the higher cellular metabolism on TCPS, BAG7.7_MEM, and BAG7.7_SHARP was also demonstrated on day 5 (Figure 3e). The ALP activity results (Figure 3f) showed that ALP activity was significantly elevated in the BAG7.7_SHARP group compared to the other groups, which was manifested by the three-times-higher ALP activity of 5.31 ± 0.58 (nmol/min/mg protein) in BAG7.7_SHARP than that in BAG7.7 (1.54 ± 0.08 nmol/min/mg protein). However, no significant difference was found between the TCPS, BAG7.7_DI water, and BAG7.7_MEM (*p* > 0.05), all of which presented higher ALP activity than BAG7.7.

### 3.2. Anti-Biofilm Properties of BAG-Loaded Resin Composites

#### 3.2.1. Cell Viability Using XTT Assay and CFU Counting

**Figure 4:**
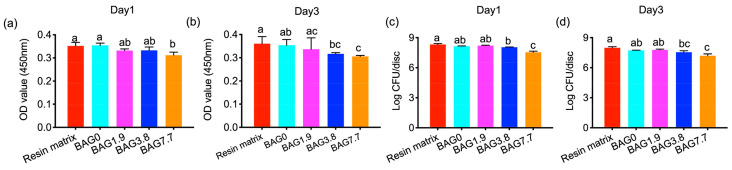
The cell viability was assessed by the XTT assay on (**a**) day 1 and (**b**) day 3, and CFU counts in bacteria biofilm on (**c**) day 1 and (**d**) day 3. Different small letters indicate significant differences between BAG groups on the same day.

The cell viability results using the XTT assay and CFU counting are shown in Figure 4. On day 1 and day 3, BAG loading lowered cell viability in a dose-dependent way, while the significantly lowest values were only found in BAG7.7, compared to the resin matrix and BAG0 (*p* < 0.05). The direct antibacterial property of experimental composites in the biofilms was assessed by counting the viable bacterial colonies, as shown in Figure 4c,d. Increasing the incorporation of BAG fillers decreased the viable count when compared with the control group and composite group without BAG. In addition, BAG7.7 outperformed other groups in eliminating viable bacterial colonies (*p* < 0.05) in both 1-day-old and 3-day-old biofilm.

#### 3.2.2. Bacteria Staining and Biofilm Structure Evaluated by CLSM

**Figure 5:**
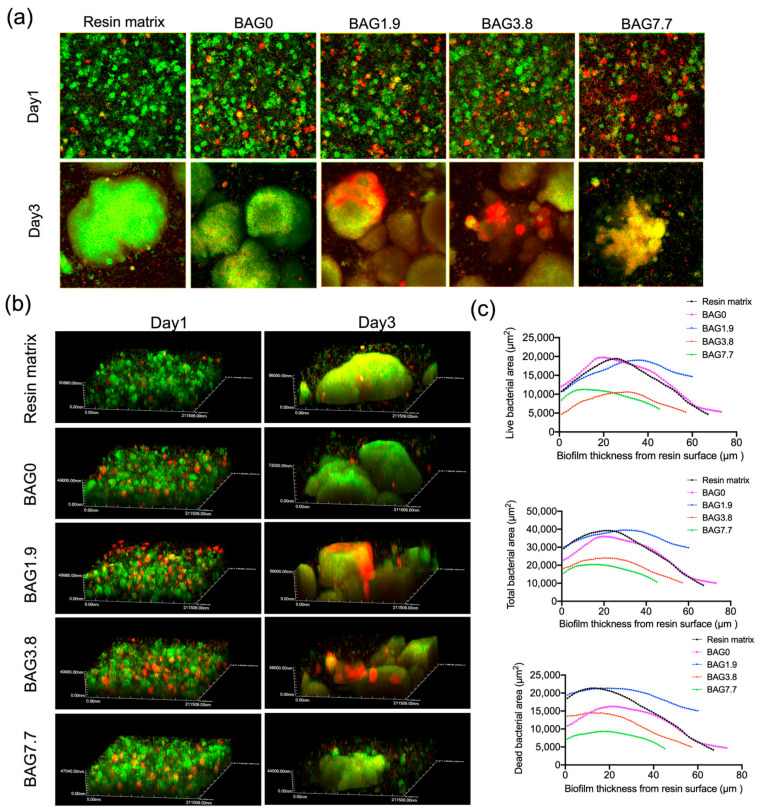
(**a**) Representative 2D live/dead staining images, (**b**) typical 3D images of *S. mutans* biofilms, (**c**) the total, live, and dead bacterial area in each layer of the 3-day-old biofilm is plotted vs. the location of the 2D image in the biofilm at distance from the specimen surface. Images are obtained at 60× magnification with an oil immersion lens.

CLSM images (Figure 5a) indicate viable and non-viable colonies (green and red, respectively) in *S. mutans* biofilm on the surface of the composites. The control groups demonstrated the most live bacteria (green colonies), whereas resins containing BAG fillers demonstrated a dose-dependent increase in dead cells at all time points. The typical CLSM images of three-dimensional (3D) biofilm on different specimens are presented in Figure 5b. Many scattered spherical bacterial clumps formed the 1-day-old biofilm structure. Additionally, the 3D images reflected that biofilms on the resin matrix were the thickest (51 µm), and on BAG7.7 they were the thinnest (47 µm) on day 1. Prolonged incubation time to day 3 achieved thicker biofilm, and biofilm thickness steadily decreased from the resin matrix (66 µm) and BAG0 (72 µm) to BAG-loaded resins, reaching a thickness of 59 µm on BAG1.9, a thickness of 56 µm on BAG3.8, and a minimum thickness of 44 µm on BAG7.7. In addition, a mushroom-cap-shaped bacterial clump was observed on the resin matrix, as opposed to the small and dispersed particles on BAG7.7.

To explore the bacteria viability distribution throughout the depth of the biofilm, the 3-day-old biofilm was further analyzed because of its distinct discrepancy among BAG groups. Figure 5c depicts the total bacterial area, live bacterial area, and dead bacterial area in each layer of the 3-day-old biofilm vs. biofilm thickness for different BAG groups. The area under the curves and the peak positions of the curves are demonstrated in Table 1. All groups demonstrated an initial ascent, then a peak, followed by a drop in the bacterial areas. The resin matrix, BAG0, BAG1.9, and BAG3.8 possessed higher total bacterial volumes than BAG7.7 with a bacterial volume of 78 × 104 μm^3^. This phenomenon was also reflected in the live bacterial volume and dead bacterial volume. Furthermore, the peak position of the total bacterial curve was 20 μm for the resin matrix, BAG0, BAG1.9, and BAG3.8, while that of BAG7.7 was the smaller (16 μm). BAG7.7 also showed the smallest peak position value of 11 μm in the live bacteria curve.

#### 3.2.3. Biofilm Morphology Evaluated by SEM

**Figure 6:**
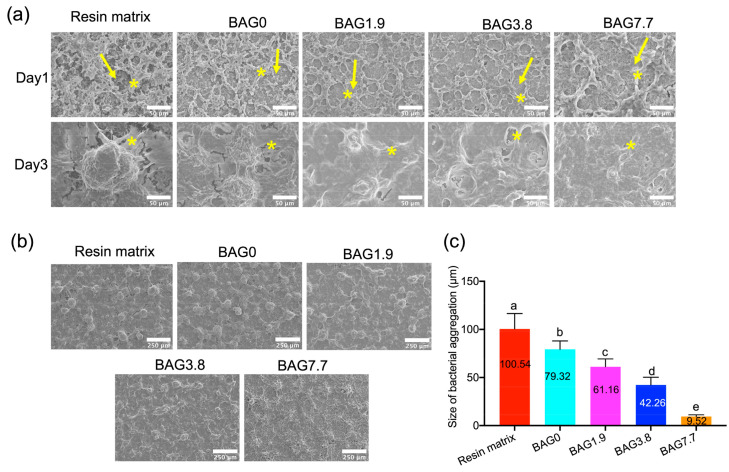
(**a**) SEM images of the morphology of biofilm growth on the surface of experimental composites on day 1 and day 3 (the yellow arrows demonstrate individual bacterial cells. The stars show extracellular matrix-like structures), (**b**) representative images of biofilm morphology (at 100× magnification), and (**c**) sizes of bacterial aggregations as determined by ImageJ in 3-day-old biofilms. The different small letters indicate significant differences between BAG groups.

The morphology of the *S. mutans* biofilms formed on the freshly cured specimens after 1 day and 3 days is presented in Figure 6. The highly organized and confluent biofilms composed of aggregated *S. mutans*, which were bridged by extracellular matrix-like structures, were demonstrated on day 1. In some areas of the biofilm surface, the cells were almost separate from the biofilm. It is noteworthy that the size of the bacteria aggregations significantly increased as the culture time increased to 3 days. The largest bacteria clumps were detected on the resin matrix. In contrast, all BAG-loaded composite groups demonstrated smaller and fewer bacterial clusters in a dose-dependent way, especially for the biofilm formed on the surface of BAG7.7. Additionally, as shown in Figure 6b,c, the aggregates in the 3-day-old biofilm generated on the control group exhibited a spherical shape of 100.54 μm in diameter, whereas those on the composites containing no BAG fillers were around 79.32 μm. As the BAG concentration increased, spherical aggregates with sizes of roughly 61.16 μm, 42.26 μm, and 9.52 μm were produced on the surfaces of BAG1.9, BAG3.8, and BAG7.7, respectively.

## 4. Discussion

The present study developed low quantities of BAG-loaded resin composites. Bis-GMA, the most common monomer in dental resins, was not used in the present study because of the proven leach of bisphenol A (BPA) into human saliva [22]. In contrast, UDMA, as a BPA-free monomer, combined with bioactive glass fillers is being employed in dental resins. It is noteworthy that unlike the weight % used for the filler content in the various studies, volume % was used to present filler loading because various fillers might have different densities and particle sizes, and the surface area to filler volume ratio directly impacts the resin viscosity and characteristics [23].

### 4.1. Effects of BAG Fillers on the Cellular Activity of Dental Resin Composites

When BAG-loaded composites meet biological fluids, it is important to consider the potential cytotoxicity due to the undesired changes in localized pH and ion concentration [7,9]. In the current study, the results demonstrated that 14-day extracts of the BAG-containing composites showed identical effects in reducing the MC3T3-E1 cell viability at all culture times, compared with BAG0 (Figure 1). The cell metabolic activity was also significantly reduced under direct exposure to the 7.7 vol% BAG-loaded composites after 3 days and 5 days of cell culture, compared with the other specimens (Figure 2). The potential cytotoxicity of experimental composites containing different amounts of BAG fillers was primarily attributed to the release of Si products and the elevated pH caused by the mineralization ability of BAG in composites, as shown in our previous study [7]. That study revealed that changes in ions and pH were time-dependent and BAG-loading dose-dependent. This is why the negative effects on cell viability were more pronounced when exposed to 14-day extracts for composites containing higher BAG amounts. Specifically, BAG7.7 induced a Si concentration of about 25 mg/L and a pH value of around 9 after 14 days of immersion in 5 mL MEM, which contributes to the lowest cellular activity among BAG groups. It is reasonable to conclude that the localized ion concentration and pH above the cells that were adherent to the resins were even higher, which caused unfavorable effects on cell viability. Based on an early study [24], Hench et al. proved that Si concentrations of 17–21 ppm are beneficial to enhancing osteoblast cell activity. However, higher Si ionic concentrations in BAG extractions have an inhibitory effect on cell proliferation [25].

ALP activity is the most used early marker of osteogenic differentiation and is utilized to determine the ability of the cells to deposit mineralized tissue [26]. As shown in Figure 1d, extracts from BAG1.9 and BAG3.8 demonstrated decreased ALP activity, compared to TCPS and BAG0, which is consistent with the cell viability results. This may be related to the inhibition potential of extracts consisting of Si products and the elevated pH, as described before. Interestingly, when BAG fillers were added at a concentration of 7.7 vol%, ALP expression increased dramatically, while it still remained lower than the control group. A study [27] demonstrated that higher concentrations of Si induced higher differentiation-related gene expression upregulation. The higher release of Si in BAG7.7 than in BAG1.9 and BAG3.8 might account for the increased ALP activity, which was still lower than that of the control group without BAG fillers due to the adverse effects of the alkaline environment. In addition, it should be noteworthy that when seeding cells on BAG resins directly, the ALP activity on BAG composites decreased in a dose-dependent manner with the lowest ALP expression in BAG7.7. This implies that the inhibition potential of BAG-loaded composites on cell viability plays a dominant role in cell differentiation when seeding cells directly on resin composites. It can be suggested that the balance of the ionic concentration between cell viability and cell differentiation needs further investigation, especially for the BAG-loaded composites.

The above findings indicate that the BAG-loaded resin composites might cause a problem in cell studies when using standard (static) cell culture methods employing both extract- and contact-exposure models due to the elevated pH and ions concentrations. However, glass degradation in vivo arises in dynamic conditions, which results in the continuous dilution and elimination of the toxic dissolution products from the material [16,17]. Accordingly, to evaluate the behavior of cells in contact with BAG-loaded resin composites in vitro, some preconditioning treatment must be employed to prevent such non-realistic toxicity from simulating in vivo conditions. Based on our previous work [7], only BAG7.7 produced the dense biomimetic calcium phosphate layer on the experimental composites after preconditioning in DI water, MEM, and the SHARP solution for 14 days. In the present study, the cellular activity of the BAG7.7 experimental composites after a one-step conditioning treatment was further assessed. Cell proliferation was highly enhanced on BAG7.7_DI water, BAG7.7_MEM, and BAG7.7_SHARP. These findings indicated that BAG-loaded resins were “more biocompatible” after 14 days of immersion in different solutions due to the elimination of the alkaline pH, released ions, and residual monomers. Similarly, in Neel et al.’s work [28], human mesenchymal stem cells were unable to adhere to calcium phosphate cement without preincubation. In contrast, the calcium phosphate cement preconditioned with supplemental growth medium and serum protein could eliminate the breakdown products that were found within the first 24 h and imitate the protein-rich in vivo environment. In the current study, thanks to the treatment in the MEM and SHARP solutions, the surface of the composites developed octacalcium phosphate (OCP) and carbonated hydroxyapatite (CHA) crystal layers [7], respectively, which could favor cell proliferation and differentiation. Furthermore, a significantly higher level of ALP activity was detected on BAG7.7_SHARP (Figure 3) compared with other groups, which was again attributed to the formation of bone-like CHA crystals, possessing good biocompatibility and strong bone guidance [29,30]. These results imply that BAG-loaded composites might possess good cellular viability and cell early differentiation in vivo.

### 4.2. Antibacterial Properties of BAG-Loaded Dental Resin Composites

The XTT assay and CFU counting demonstrated the antibacterial effects of experimental BAG-containing resin composites when compared with pure resin and control composites (Figure 4). This was especially reflected in BAG3.8 and BAG7.7. Furthermore, live/dead staining (Figure 5a) revealed that the increase of BAG fillers loading in resin composites increased non-viable bacteria. Based on our prior study [7], the introduction of 1.9 vol% BAG fillers contributed to the release of Si (1.5–5.4 mg/L) into the immersion solutions, which was slightly higher than those containing no BAG (1.1–3.4 mg/L), with no significant difference in pH changes (7.0–7.4). This implies that the inhibition action of released Si may be the key to the antibacterial properties in the current study. The research demonstrates that the antibacterial mechanism of ions can be explained by the production of reactive oxygen species and the interactions of these ions with the cell membrane, causing damage to the proteins and DNA of bacteria [31]. In the current study, the release of Si was more evident for the higher BAG loading, especially for resin composites containing 7.7 vol% BAG. The Si released from BAG7.7 was 10–35 mg/L, which was 10 times higher than that released from the unmodified resin composites, which explains the greatest antibacterial performance in BAG7.7. BAG7.7 also presented the highest pH values in the range of 8.5–9.2, compared to BAG0; however, this fell within the *S. mutans*-tolerated pH range of 4–10 as documented in the literature [32]. In the previous study, the pH measurement was conducted in a 5 mL immersion solution, while the bacteria were cultured on BAG-loaded resins directly with only 400 μL BHI broth in the current study. This implies that the antibacterial effects coming from the alkaline microenvironment may be unveiled when bacteria were cultured on resins directly, even with a daily refresh of culture media.

Unlike planktonic bacteria, biofilm development increases resistance to unfavorable environmental impacts, such as antibiotics and antimicrobial agents. In the current study, the anti-biofilm efficacy of experimental BAG-loaded resin composites, especially in the alterations in biofilm thickness, as well as bacteria viability throughout the biofilm and biofilm morphology, were visualized for the first time via CLSM 3D reconstruction and SEM. As shown in Figure 5b, BAG-loaded composites led to a decline in biofilm thickness, particularly for the 3-day-old biofilm, which presented a decreased thickness of 59 µm for BAG1.9, 56 µm for BAG3.8, and the smallest thickness of 44 μm for BAG7.7, respectively.

On the one hand, this phenomenon is related to the bactericidal impact of an alkaline environment and ionic osmotic pressure. The evidence of biofilm inhibition was further provided from the SEM analysis, which presented a significant reduction of surface coverage and dispersion of bacteria in 3-day-old biofilm on higher BAG loading, especially on BAG7.7. In addition, an earlier drop in the total bacterial area on BAG7.7, together with the live bacterial area, also verified that BAG7.7 could inhibit bacteria close to the composite surface. Similarly, S53P4 bioglass has the potential to reduce biofilm mass by around 80% when compared to negative controls, regardless of the bioglass formulation. However, the introduction of dynamic liquid systems would most likely have lowered the anti-biofilm activity reported [33]. Based on the above results, it can be speculated that a higher BAG loading of more than 7.7 vol% may endow the greater anti-biofilm effects. However, the mechanical properties may be compromised when incorporating higher than 20 wt% (7.7 vol%) BAG fillers due to the unfavorable influence of BAG on the curing potential, according to our literature review [34]. In addition, the higher alkaline environment and osmotic pressure may achieve cytotoxicity. This is why we used the maximum BAG concentration of 7.7 vol% to modify the resin composites in this study.

## 5. Conclusions

In summary, multi-functional bio-safe UDMA-based composites containing low quantities of BAG were developed. Even though the experimental composites demonstrated impaired cell viability with the increase in BAG loading in the static experimental conditions, using MEM and SHARP solutions to precondition BAG-loaded resin composites led to unimpaired or even better cell adhesion, cell proliferation, and early cell differentiation. Furthermore, the incorporation of BAG fillers not only reduced cell viability in *S. mutans* biofilm on the composite surface but also demonstrated anti-biofilm properties, including the suppression of biofilm thickness, bacterial volume, and bacterial aggregation. This phenomenon was more evident in experimental composites containing higher BAG, particularly BAG7.7, because the dissolution of BAG contributes to the highest ionic osmotic pressure and alkaline environment. These experimental resin composites seem to be promising for preventing caries and effectively treating dental defects in vivo.

## Figures and Tables

**Table 1:** The area under the curves and the peak positions of total bacterial curves, live bacterial curves, and dead bacterial curves of 3-day-old biofilms on the resin matrix, BAG0, BAG1.9, BAG3.8, and BAG7.7.

	Area under Curves (×10^4^ μm^3^)			Peak Position (μm)		
	Total Bacteria	Live Bacteria	Dead Bacteria	Total Bacteria	Live Bacteria	Dead Bacteria
Resin matrix	191	91	100	21	25	13
BAG0	185	100	85	20	20	22
BAG1.9	210	96	114	29	36	16
BAG3.8	110	47	63	20	30	11
BAG7.7	78	43	36	16	11	18

## Data Availability

The data that support the findings of this study are available upon request. The data are not publicly available due to privacy restrictions.
